# What factors affect the methodological and reporting quality of clinical practice guidelines for osteoporosis? Protocol for a systematic review

**DOI:** 10.1097/MD.0000000000021811

**Published:** 2020-08-14

**Authors:** Peng-Zhong Fang, Ya-Min Chen, Jin-Lei Chen, Jun-Hao Sun, Jian-Shi Tan, Rui-Rui Wang, Xin Wang

**Affiliations:** a The First Clinical Medical College of Lanzhou University, Lanzhou City; b Evidence-Based Nursing Center, School of Nursing, Lanzhou University; c Department of Orthopedics, The First Hospital of Lanzhou University, Lanzhou, Gansu Province, China.

**Keywords:** osteoporosis, clinical practice guidelines, quality, recommendations

## Abstract

**Background:**

Osteoporosis is a disease with a high prevalence and low treatment rate, which poses a serious threat to the lives of patients and brings a heavy economic burden. Clinical practice guidelines (CPGs) provide vital guidance for disease management. Up to now, different countries, regions, and organizations have issued a certain number of CPGs for osteoporosis, but the recommendations in different guidelines are inconsistent. This protocol plans to evaluate the quality of the CPGs for osteoporosis and then make a comparative analysis of the recommendations in the CPGs.

**Methods:**

Several databases including PubMed, Web of Science, Embase, and Cochrane Library, as well as the official website of relevant organizations will be searched. Screen and data extraction will be performed by two reviewers independently, and the third reviewer help to resolve the divergence between them. Using the AGREE II instrument and RIGHT checklist to assess the methodological and reporting quality of the CPGs. The extracted recommendations, including but not limited to screening, diagnosis, evaluation and treatment, will be summarized and analyzed, and the results will be presented in tabular form. Bubble charts will be used to show quality differences between CPGs and to describe the correlation between methodological and reporting quality through regression analysis. Excel, EndnoteX9 and SPSS 25.0 will be used.

**Result:**

To evaluate the advantages and disadvantages of the existing CPGs of osteoporosis and analyze the similarities and differences between the recommendations, the results will be published in a peer-reviewed journal.

**Conclusion:**

This study will provide systematic evidence for existing CPGs of osteoporosis and to provide a reference for CPGs users.

**Protocol Registration:**

INPLASY 202070031.

## 1 Introduction

Osteoporosis is a common systemic skeletal disorder, which is characterized by bone mass loss and fragility fracture.^[1,2]^ It mainly affects postmenopausal women, while osteoporosis in men is also a health problem that cannot be ignored.^[3]^ In the world, 18.2% of women and 3.1% of men over 50 years old tend to fracture in 2010, with the increasing of the world's total population and the aggravation of population aging, it will double by 2040.^[4]^ In 2010, the overall prevalence of osteoporosis in American community residents was expected to be 10.3%, and low bone mass is about 43.9%, which affected 53.6 million (54%) of the elderly in the United States.^[5]^ 0.83% of the world's burden of non-infectious diseases is caused by osteoporotic fractures.^[6]^ In Europe, the medical expenses of osteoporosis and related fractures exceeded 39 billion euros per year.^[7]^ It is estimated that by 2025, the annual direct medical expenses of osteoporosis-related fractures will reach $25.3 billion in the United States.^[8]^

Osteoporosis is the result of multiple factors such as heredity, nutrition, aging, hormone level, and so on.^[9]^ Its systematic management measures include early prevention and diagnosis, drug treatment, lifestyle intervention and nursing. Drug therapy is the most important measure to increase bone strength. At present, there are a variety of drugs with different mechanisms for the treatment of osteoporosis.^[10]^ Fragility fracture is the outcome of osteoporosis, and fracture risk is positively correlated with age and elderly hip fracture has high disability and mortality rate,^[2,11]^ which is called as “the last fracture in life”. Falling is the most direct cause of fractures. Therefore, preventing falls is sometimes better than osteoporosis to reduce the incidence of fractures.^[12]^ The development of the fracture risk assessment tool (FRAX) has made up for some of the limitations of bone mineral density(BMD) based diagnosis, and can more effectively screen individuals with high fracture risk.^[13]^ However, osteoporosis has a slow onset, concealment, and fewer patients with typical clinical symptoms.^[14]^ Before a fracture occurs, some patients may not receive the assessment and treatment of osteoporosis because of several factors, including education level, economic factors and marital status.^[15]^

CPG is the best guidance for interventions after a systematic evaluation of available evidence and consideration of other favorable or adverse factors,^[16]^ which is the main reference for disease management. It is fundamental to the standardized management of diseases, and the accurate implementation of health policies. The published guidelines for osteoporosis vary in quality and the recommendations are inconsistent. For example, CPG published by National Osteoporosis Foundation in 2014 recommended BMD testing should be performed in men age 70 and older.^[17]^ But, US Preventive Services Task Force guidelines issued in 2018 consider that current evidence is insufficient to support screening for osteoporosis in men.^[18]^ Another example, American College of Physicians guideline published in 2017 recommend that BMD monitoring should not be performed during the five-year medical treatment of osteoporosis in women.^[19]^ The Endocrine Society Guidelines published in 2020 recommend that postmenopausal women with low bone density and high risk of fractures be monitored BMD by dual-energy X-ray absorptiometry (DXA) every 1 to 3 years to evaluation of curative effect.^[20]^ Different recommendations on the same issue may bring confusion to users of the guidelines. Therefore, it is necessary to assess the quality of osteoporosis CPGs and analyze the recommendations.

## 2 Methods

### 2.1 Study design

This study plans to use the AGREE II instrument and RIGHT checklist to evaluate the methodological and reporting quality of CPGs of osteoporosis already published, calculate the correlation between them using regression analysis and compare the recommendations in the guidelines.

### 2.2 Study registration

As this is a literature-based review, ethical approval is not required. This study has been registered in the International Platform of Registered Systematic Review and Meta-analysis Protocols (INPLASY) database (protocol number: INPLASY 202070031, DOI:10.37766/inplasy2020.7.0031). This protocol will be reported complies with Preferred Reporting Items for Systematic Reviews and Meta-Analyses (PRISMA-P).^[21]^

### 2.3 Data sources and search strategy

This study will search the following databases: PubMed, Web of Science, Cochrane Library, EMBASE, Chinese biomedical literature database (CBM). Besides, the National Institute for Health and Clinical Excellence (NICE, https://www.nice.org.uk), International Osteoporosis Foundation (IOF, https://www.iofbonehealth.org), Scottish Intercollegiate Guidelines Network (SIGN, https://www.sign.ac.uk), Guidelines International Network(GIN, https://www.g-i-n.net), Yimaitong website(http://www.medlive.cn), Chinese Medical Association (CMA, https://www.cma.org.cn) will be Searched. Related words will be searched by medical subject headings (Mesh) or text word search. The retrieval strategy will be designed for each database. The language is limited to English and Chinese, and there are no other restrictions. The retrieval strategy of PubMed is shown in Table 1. When the screening is completed, check the references list of included CPGs to determine whether other CPGs can be included.

**Table 1:**
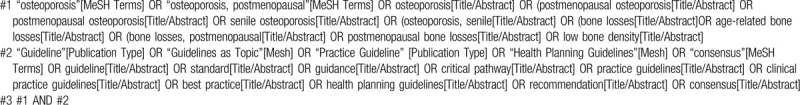
Searching strategy in PubMed.

### 2.4 Eligibility criteria

#### 2.4.1 Inclusion criteria

(1) Issued in the form of guidelines or recommendations
(2) Mainly for osteoporosis, which involves screening, assessment, diagnosis, treatment or management
(3) Language is limited to English and Chinese
(4) If there is an updated relationship, the latest version is included.

#### 2.4.2 Exclusion criteria

(1) Mainly for secondary osteoporosis, adolescent idiopathic osteoporosis or specific diseases status, such as breast cancer, gastrointestinal disease, liver disease
(2) Traditional Chinese medicine CPGs
(3) Consensus documents or position statement documents
(4) Executive summary of the guidelines or translation version based on the original
(5) Guidelines developed by individuals.

### 2.5 Measured outcomes

Methodological and reporting quality assessments of osteoporosis CPGs will be conducted to determine the evaluation results for each field and entry, and statistical analysis will be performed. The recommendations in CPGs, including screening, assessment, diagnosis and treatment, etc., will be integrated to analyze their consistency and inconsistency.

### 2.6 Determination of eligibility

**Figure 1:**
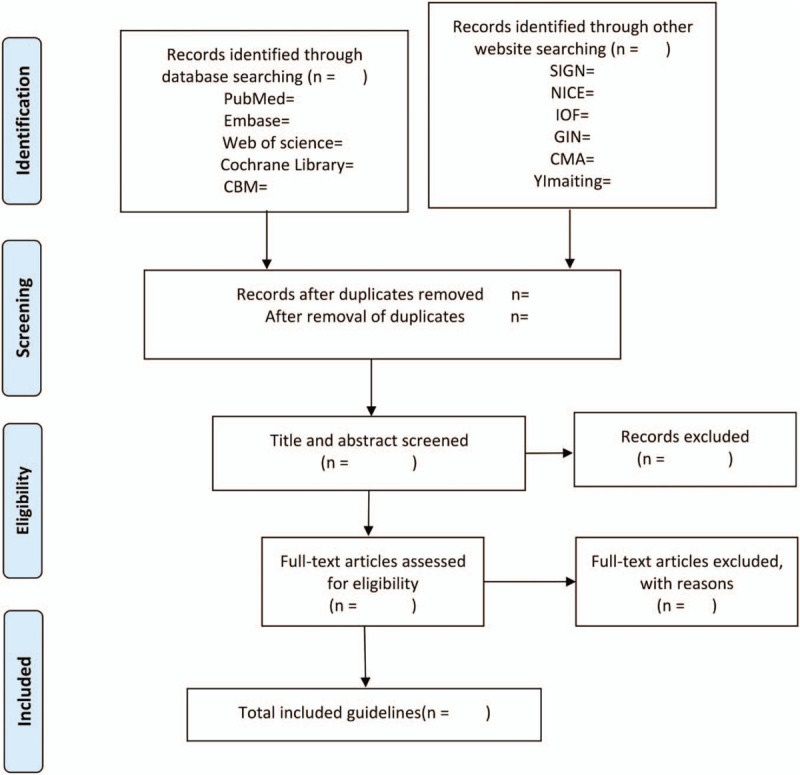
The process of study selection. CBM = Chinese Biomedical Literature Database, SIGN = Scottish Intercollegiate Guidelines Network, NICE = National Institute for Health and Clinical Excellence, IOF = International Osteoporosis Foundation, GIN = Guidelines International Network, CMA = Chinese Medical Association. From: Moher D, Liberati A, Tetzlaff J, Altman DG, The PRISMA Group (2009). Preferred Reporting Items for Systematic Reviews and Meta-Analyses: The PRISMA Statement. PLoS Med 6(7): e1000097.

All the searched records will be imported into EndnoteX9 and screened by 2 reviewers independently. Duplicate entries will be removed, and irrelevant literature will be excluded through reading titles and abstracts. The records that need to read the full text will be reviewed according to the inclusion and exclusion criteria and the reasons for exclusion will be recorded, the controversial literatures will be discussed with the third reviewer to decide whether to include them. The screening process will be shown through the PRISMA flow chart (Fig. 1).

### 2.7 Data extraction

Two reviewers pre-extracted the data of 3-5 CPGs through the pre-set data extraction table and revised it according to the problems encountered.

The content of the data to be extracted includes:

1. General information:
  a. Title and subtitle
  b. Name of the first author
  c. Number of authors
  d. Year of publication
  e. Whether it is an updated version
  f. Type of CPGs(such as screening, diagnosis, treatment)
  g. Country or region
  h. Organization
  i. Target user
  j. Target population.
2. Methodological information:
  k. Types of studies included
  l. Evidence classification and evaluation criteria
  m. Recommendations formation method
  n. Recommendations classification standard
  o. Funding sources
  p. Update plan
  q. Peer review.
3. Recommendations:
  r. Contents of recommendations
  s. Strength of recommendation
  t. Quality of evidence.

### 2.8 Quality Appraisal

The AGREE II instrument is the most widely used methodological quality assessment tool for CPGs which was published in 2003^[22]^ and revised in 2009.^[23]^ It contains 23 items in 6 fields, and each item has a corresponding evaluation standard.^[24]^ The specific content can be accessed at https://www.agreetrust.org. The RIGHT working group released the RIGHT reporting checklist in 2006, which contains 22 items in 7 areas. It is mainly used to assist in the development of CPGs and evaluate the quality of reports.^[25]^ The specific contents of the RIGHT reporting checklist can be accessed at http://www.right-statement.org.

At least 2 reviewers independently evaluate the quality of CPGs use the AGREE II instrument and RIGHT checklist. The reviewers are trained in advance, independently evaluate the 4 CPGs in other fields, and discuss the evaluation results to reach a unified standard. The reviewers score each item of AGREE II with 1-7 points: a score of 7 means complete agreement and 1 point to the contrary. The RIGHT list can be evaluated as the report (Y), unreported (N), partial report (P) and not applicable (NA).^[26]^ AGREE II scoring items with a difference of more than 2 points or inconsistent RIGHT items evaluations will be discussed again with the third reviewer.

### 2.9 Data synthesis

The intra-class correlation coefficient (ICC) will be used to assess the differences between reviews and ICC ≥ 0.7 indicated that there is a small difference. The scaled domain score of AGREE II for each CPGs will be calculated.^[22]^ The range, average, standardization deviation and median of standardization percentage in each field will be counted. The reported percentage of each item in the RIGHT checklist of all CPGs and the reported number and percentage of all items in each CPGs will be performed the statistical analysis. Regression analysis used to calculate the correlation between methodological and reporting quality.

All recommendations will be extracted and comprehensively analyzed to clarify their consistency and inconsistency and to analyze the reasons for the inconsistency. Bubble charts will be used to show the differences in methodological and reporting quality between CPGs. Tables or mind maps will be used to show the results of the recommendation analysis. Whether to conduct a subgroup analysis is based on the results of data extraction. The statistical processes will be performed by Excel and SPSS 25.0.

## 3 Discussion

With the standardization of disease diagnosis and treatment and the transformation from empirical medicine to evidence-based medicine, clinical practice guidelines become indispensable because of its guiding role in clinical practice. The aging population has led to an increasing prevalence of osteoporosis. High-quality CPGs are needed for osteoporosis management. However, there are differences in the quality of the guidelines, and there are often opposite recommendations on the same clinical problem, which has confused the user's choice of CPGs. This study is firstly to combine the AGREE II instrument with the RIGHT checklist to assess the quality of osteoporosis CPGs. It will also conduct a comprehensive analysis of the recommendations in different guidelines, including screening, prevention, diagnosis, treatment, and so on. The results of the study will show the advantages and disadvantages of the current osteoporosis CPGs in methodology and report quality, and provide a reference for users to select guidelines and benefit for future research. However, the research is limited to including only English and Chinese guidelines, mainly for elderly primary osteoporosis, and there is no evaluation of other types of osteoporosis guidelines.

## Acknowledgment

We thank Dr Jinhui Tian for his guidance on the methodology of this study.

## Author contributions

PZF, YMJ, and XW conceived this study. PZF, YMC, and XW designed the inclusion/exclusion criteria and the searching strategy. JLC and JHS designed a data extraction table. PZF and YMC will be searched for the literature. PZF, YMC, and JHT will be collected the data and made statistical analysis. PZF, RRW, and XW drafted the protocol and revised the manuscript.
